# Bionomics and insecticide resistance of the arboviral vector *Aedes albopictus* in northern Lao PDR

**DOI:** 10.1371/journal.pone.0206387

**Published:** 2018-10-25

**Authors:** Julie-Anne A. Tangena, Sébastien Marcombe, Phoutmany Thammavong, Somsanith Chonephetsarath, Boudsady Somphong, Kouxiong Sayteng, Marc Grandadam, Ian W. Sutherland, Steve W. Lindsay, Paul T. Brey

**Affiliations:** 1 Medical Entomology and Vector-Borne Disease Laboratory Institut Pasteur du Laos, Vientiane, Laos; 2 Arbovirology and Emerging Viruses Laboratory, Institut Pasteur du Laos, Vientiane, Laos; 3 United States Naval Medical Research Center—Asia, PSA SEMBAWANG, Singapore; 4 United States Navy Entomology Center of Excellence, NAS Jacksonville, Florida, United States of America; 5 Department of Biosciences, Durham University, Durham, United Kingdom; Centro de Pesquisas René Rachou, BRAZIL

## Abstract

In the last four decades there has been a staggering increase in the geographical range of the arboviral vector *Aedes albopictus* (Skuse, 1894). This species is now found in every continent except Antarctica, increasing the distribution of arboviral diseases such as dengue and chikungunya. In Lao PDR dengue epidemics occur regularly, with cases of chikungunya also reported. As treatment methods for arboviral diseases is limited, the control of the vector mosquitoes are essential. There is a paucity of information on the bionomics and resistance status of this mosquito for successful vector control efforts. Here we describe the bionomics and insecticide resistance status of *Ae*. *albopictus* in Laos to identify opportunities for control. Adult *Ae*. *albopictus* were collected using human-baited double bed net (HDN) traps in forests, villages and rubber plantations and tested for alpha- and flaviviruses with RT-PCR. Surveys were also conducted to identify larval habitats. Seven adult and larval populations originating from Vientiane Capital and Luang Prabang province were tested against DDT, malathion, permethrin, deltamethrin and, temephos following WHO protocols. *Aedes albopictus* were found throughout the year, but were six-fold greater in the rainy season than the dry season. Adult females were active for 24 hours, with peak of behaviour at 18.00 h. The secondary forest and rubber plantation samples showed evidence of Pan-flaviviruses, while samples from the villages did not. More than half of the emerged *Ae*. *albopictus* were collected from mature rubber plantations (53.9%; 1,533/2,845). Most *Ae*. *albopictus* mosquitoes emerged from latex collection cups (19.7%; 562/2,845), small water containers (19.7%; 562/2,845) and tyres (17.4%; 495/2,845). Adult mosquitoes were susceptible to pyrethroids, apart from one population in Vientiane city. All populations were resistant to DDT (between 27–90% mortality) and all except one were resistant to malathion (20–86%). Three of the seven larval populations were resistant to temephos (42–87%), with suspected resistance found in three other populations (92–98%).This study demonstrates that rural areas in northern Laos are potential hot spots for arboviral disease transmission. Multiple-insecticide resistance was found. *Aedes albopictus* control efforts in villages need to expand to include secondary forests and rubber plantations, with larval source management and limited use of insecticides.

## Introduction

Over the past forty years *Aedes albopictus* (Skuse, 1894) has expanded its geographical range from the rainforests of South-East Asia (SEA) to every continent except Antarctica [1–3], and has contributed to the spread of dengue and chikungunya viruses (CHIKV) around the tropics and sub-tropics, particularly in rural areas [3–6]. *Aedes albopictus* is an important secondary vector of dengue and chikungunya [7]. It may also be a potential vector of Zika, although this is still in early stages of investigation [8–10]. Outbreaks of dengue associated with *Ae*. *albopictus* have occurred in Africa, China, East Asia, Europe, Pacific and USA [4, 11–17]. The CHIKV has recently adapted to *Ae*. *albopictus* [18–20], resulting in outbreaks in the Caribbean, Indian Ocean and southern Europe [2, 21–24].

The range expansion of this species is associated with the increase in the global trade in used tyres and lucky bamboo [25, 26], and the transportation of the drought-resistant eggs by air and sea traffic around the world [25–27]. The mosquito is flexible in its larval habitats, host preference and place of feeding, and can readily adapt to new environments in both tropical and temperate areas [28, 29]. Generally, the species is most common in suburban, rural and forested areas [28], but can also occur in highly dense urban areas [7, 30]. The immature stages are mostly found in indoor artificial containers closely associated with human dwellings [31]. They feed almost entirely on humans, mainly during daylight hours, both indoors and outdoors. Typically, these mosquitoes do not fly far, remaining within 100m around their breeding site. In the absence of a fully effective vaccine against dengue [32], and no specific treatments for the control of DENV and CHIKV, vector control strategy in Lao PDR relies heavily on insecticides [33–35].

*Aedes albopictus* is one of the most common mosquitoes in Lao PDR [36, 37], yet its role in the transmission of arboviruses in the country is unclear. In the last decade, there have been outbreaks involving all four DENV serotypes, both in rural and urban areas [38–42]. The most recent outbreak was in 2017 with 18,000 syndromic cases reported [43]. Likely both *Aedes aegypti* and *Ae*. *albopictus* were involved in the outbreak, although studies are absent [36, 37]. Co-circulation of dengue with chikungunya and with Japanese encephalitis has been identified [44, 45]. Little is known about chikungunya disease dynamics in Lao PDR. Antibodies against CHIKV were first detected in 1966 [46], with the presence of the virus not detected until 2012 [47]. The incidence of DENV and CHIKV are becoming more common in Lao PDR with active circulation of dengue within SEA [48], highlighting that favourable conditions for their transmission exists. These diseases will continue to be important health concern for the region [38, 45, 48–50]. The control of arboviral diseases in Lao PDR depends on vaccines for Japanese encephalitis, early alert systems using appropriate diagnosis, and mostly on the use of insecticides in areas where people live. There is a need to include additional vector control methods to maintain sustainability of public health intervention programs. A deeper understanding of the bionomics of *Ae*. *albopictus* might provide opportunities.

Resistance to the main classes of insecticides used in public health for vector control have been recorded around the world, including in South-East Asia (SEA) [51–55]. Resistance of *Ae*. *albopictus* populations to DDT, dieldrin and fenthion were first identified in the region in the 1960s [56]. More recent studies revealed that *Ae*. *albopictus* populations in SEA were also resistant to organophosphates, pyrethroids or both [57–62]. To our knowledge, no *Ae*. *albopictus* insecticide susceptibility studies have been conducted in Lao PDR. DDT has been used in the country from the 1950s, for agriculture and vector control, until it was banned in 2010 [63]. The organophosphates malathion and temephos have been used since the 1990s and pyrethroids such as deltamethrin and permethrin, have been used for long-lasting insecticidal nets (LLINs), thermal fogging and indoor residual spraying (IRS) since the early 2000s. The high dependence of dengue control on insecticides and the absence of vector resistance information in the country hamper the efficiency of prevention and control strategies.

There have been no comprehensive studies in Lao PDR on the behaviour, ecology and, insecticide susceptibility of *Ae*. *albopictus* [31, 64–66]; information needed to identify effective methods for vector control interventions. In order to fulfil theses gaps, we combined bionomics data from previous studies in rural areas of Lao PDR to identify new opportunities for vector control [36, 37]. This secondary analysis highlights the bionomics of *Ae*. *albopictus* specifically. We also determined the insecticide resistance status of several *Ae*. *albopictus* populations to commonly used insecticides.The combination of data from these studies provides a comprehensive description on *Ae*. *albopictus* in Lao PDR.

## Materials and methods

### Study design

Studies on the adult and larval ecology of mosquitoes in northern Lao PDR have been described previously [36, 37]. In the study areas, *Ae*. *albopictus* was the dominant species. A second line of analysis was done on these specimen to identify new control opportunities, focussing specifically on the peak of *Ae*. *albopictus* activity between 6.00 and 18.00 h. In brief, this consisted of surveying adult and larval mosquitoes in forests, villages and rubber plantations in three study areas (Thinkeo, Silalek and Houayhoy) in Luang Prabang province, Lao PDR. In addition, we conducted insecticide susceptibility tests on adult and larval *Ae*. *albopictus* for DDT, deltamethrin, malathion, permethrin and temephos. From all three study areas (Thinkeo, Silalek and Houayhoy) mosquito collections were done for insecticide susceptibility tests. However, only from Houayhoy area enough mosquitoes were collected for analysis. Additionally, *Ae*. *albopictus* samples were collected from Vientiane city and Luang-Prabang city for representation of urban populations.

### Adult survey

**Fig 1:**
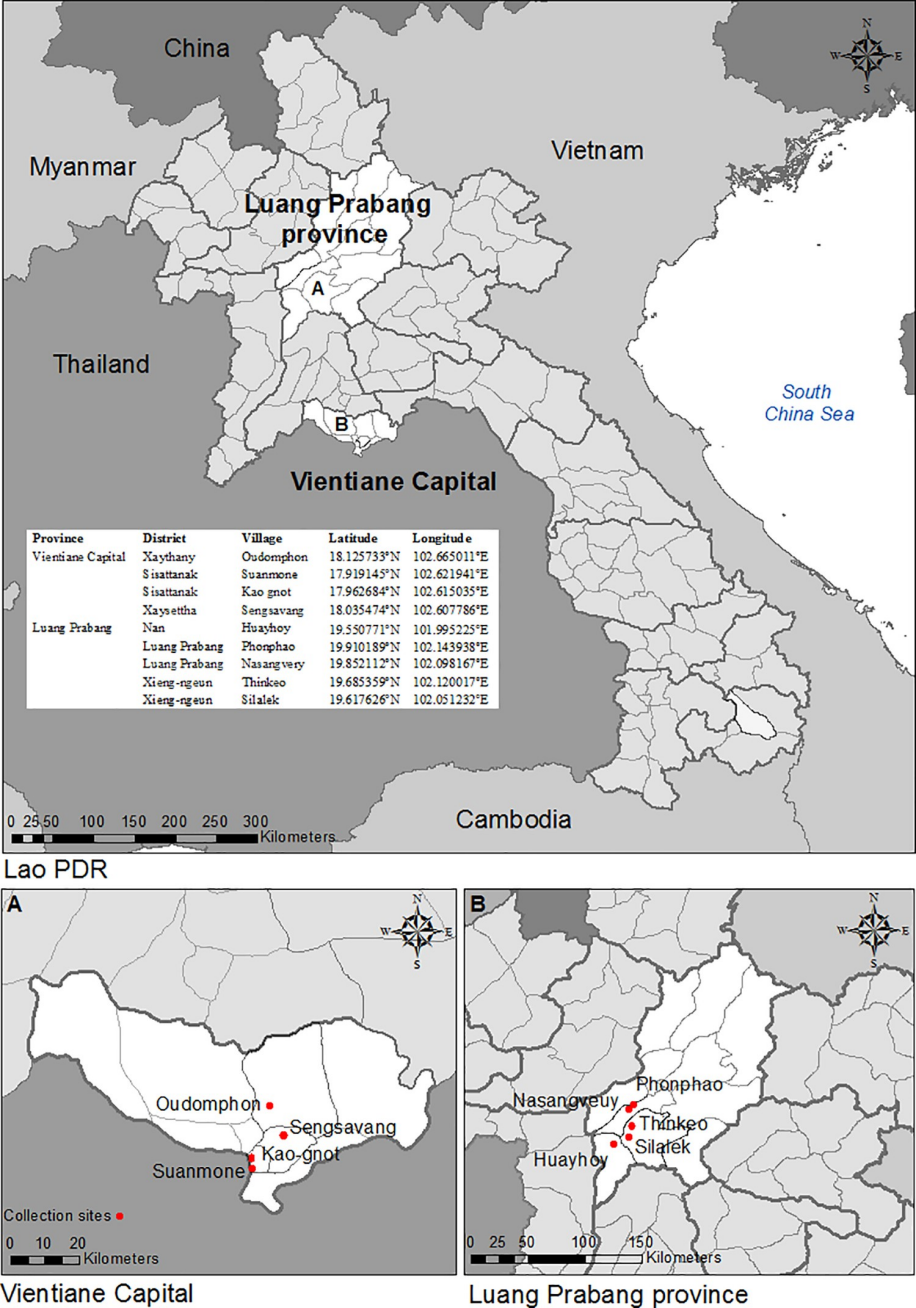
Map of Lao PDR showing the study sites in Vientiane capital and Luang Prabang provinces.

Adult mosquitoes were collected monthly from July to November 2013 and in February, March, May and, July 2014 in three study sites in Luang Prabang province, northern Lao PDR: Thinkeo, Silalek and Houayhoy (Fig 1). In each study site four rural habitats were surveyed: a secondary forest, a rural village, a mature rubber plantation and an immature rubber plantation. The secondary forests were relatively young forests with high undergrowth and canopy cover. Villages were linearly organized settlements, with most houses constructed from bamboo. The mature rubber plantations consisted of rubber trees over 10 years old that were tapped for latex. The plantations were characterized by a high density of rubber trees with high canopy cover and little undergrowth. The immature rubber plantations were those with trees less than five years old where no latex tapping occurred, with low, dense undergrowth and less canopy cover than the more mature plantations.

Mosquitoes were sampled using the human-baited double bed net (HDN) trap [67]. Three HDN traps were used in each habitat, totalling 36 HDN traps. For each trap, a participant rested on a bamboo bed covered by two untreated bed nets and collected mosquitoes from between the two nets for 10 minutes every hour. Mosquitoes were morphologically identified to species or species complex using the identification keys of Thailand [68]. Every month, in one study area 12 participants collected mosquitoes in the four different habitats simultaneously for six hours, after which they were replaced by twelve new participants. This was repeated four times during several days to collect 48 h of monthly data in each habitat. Therefore, in each of the three study areas a total of 24 participants and two supervisors between 18 and 55 years old participated in the study (n = 78), and gave informed written consent.

Parity was determined from specimens collected using two HDN traps in the four different habitats of Thinkeo study area (Fig 1) during the rainy season, in July and August 2015. Collections were done from 17.00–06.00 h on 42 nights. The ovaries of the female mosquitoes were dissected to determine the percentage of parous mosquitoes; mosquitoes that have laid eggs before [69].

### Molecular identification of arboviruses

Adult female *Ae*. *albopictus* were tested for the presence of alphavirus and flavivirus sequences. The abdomen, wings and legs of *Ae*. *albopictus* samples were pooled, with a maximum of 10 samples per tube. Pools were separated into males and females, habitat type and month of collection. Blood-fed mosquitoes were analysed individually, to be able to discriminate an infected mosquito from a contaminated blood meal. RNA was extracted using the NucleoSpin® 8 Virus (Ref: 740 643.5) extraction kit and amplified using specific primers with RT-PCR for the nested PCR (external primers Alpha1- KYT CYT CIG TRT GYT TIG TIC CIG G, Alpha1+ GAY GCI TAY YTI GAY ATG GTI GAI GG and the internal primers Alpha 2- GCR AAI ARI GCI GCY TYI GGI CC, Alpha 2+ GIA AYT GYA AYG TIA CIC ARA TG; external primers Flavi1- TCC CAI CCI GCI RTR TCR TCI GC, Flavi1+ GAY TYI GGI TGY GGI IGI GGI RGI TGG and internal primers Flavi 2- CCA RTG ITC YKY RTT IAI RAA ICC, Flavi2+ YGY RTI YTY AWC AYS ATG GC) [70, 71] and screened for the alphavirus (195 base pair) and flavivirus (143 base pair) genome sequence using agarose gel electrophoresis. Chikungunya, Metri and Sindbis virus were used as positive controls for Pan-alpha identification. Positive controls for Pan-flavi identification were dengue, West Nile and Japanese encephalitis virus.

### Larval survey

In 2014 larval surveys were carried out in the same area where the adult surveys were done the previous year (Fig 1), in three villages, three mature rubber plantations and three immature rubber plantations. The secondary forests were not surveyed, due to the limited resources available and difficulties accessing the areas. From August to December 2014 in each of the nine habitats, a 1 km^2^ area was surveyed monthly. All water bodies within the areas were logged with a Global Positioning System (Garmin GPS map 62sc, Garmin International Inc, Kansas, USA) and classified into one of 15 waterbody types, described in S1 Table. The presence of *Ae*. *albopictus* larvae and pupae was determined using one to ten dips (depending on the habitat size) with a 350 ml standard dipper (Bioquip, California, USA). If immature mosquitoes were found, dipping was continued for an additional 10 minutes to collect samples. Immature mosquitoes were transported to the field laboratory, reared to adults and morphologically identified to species using the Thai identification keys [68].

### Insecticide resistance

Insecticide susceptibility tests were conducted with *Ae*. *albopictus* populations collected from urban and rural areas where dengue outbreaks occurred in 2013 (Fig 1) [38]. During the rainy season of 2015, from June to September, larval and pupal collections were carried out in urban areas. Households and temples were surveyed in Luang Prabang city, in Nasangveuy village and in Phonphao village. Furthermore, during the rainy season of 2015 and 2016 mosquito larvae were weekly collected in several districts of Vientiane Capital city; Kao-gnot district, Suanmone district, Sengsavang district. Mosquitoes were also collected in rural Houayhoy village, one of the field sites of the adult survey, and in rural areas of Oudomphon village. All collection sites were geo-referenced with a GPS system (Fig 1). Immature mosquito stages were reared to adults in the laboratory on a diet of powdered cat food (Whiskas®). In the laboratory, mosquito colonies were reared using standardized techniques [54]. Larvae and adults obtained from the F_1_ or F_2_ progeny were used for bioassays, performed following the standard WHO protocols [72].

#### Adult bioassays

Adult bioassays were done by exposing female mosquitoes to filter papers treated with diagnostic doses (DD) [72] of all insecticides used for adult vector control in Lao PDR; 4% DDT, 0.05% deltamethrin, 0.8% malathion and 0.25% permethrin, obtained from the Vector Control Research Unit, Universiti Sains Malaysia. For each strain, four batches of 25 three to five day old female mosquitoes (n = 100) were exposed for one hour to the insecticides using WHO bioassay tubes [72]. Control treatments were exposed to filter papers impregnated with the insecticide carrier (silicon or risella oil). The adults were then transferred into holding tubes, were provided with sugar solution (10%), and kept at 27°C with a relative humidity of 80%. Mortality was recorded 24 h after exposure.

#### Larval bioassays

Larval bioassays were performed using late third- and early fourth-instar larvae of the F_1_ and F_2_ progenies. Larval bioassays were carried out using diagnostic doses determined at the laboratory for the *Ae*. *aegypti* USDA susceptible reference strain [73]. The insecticides DDT (0.04 mg/L), deltamethrin (0.00132 mg/L), malathion (1 mg/L), permethrin (0.0014 mg/L) and temephos (0.0132 mg/L) were tested by diluting the active ingredients (ai), purchased from Sigma-Aldrich (Seelze, Germany), in absolute ethanol to obtain the required concentration according to WHO guidelines [74]. For each bioassay, 100 larvae of each strain were transferred to four cups (n = 25 larvae/cup) containing 99 mL of distilled water and 1 mL of the insecticide at the desired concentrations. Control treatments were made with 99 mL of distilled water and 1 mL of ethanol. Mortality was recorded after 24 hours.

### Statistical analysis

For the adult *Ae*. *albopictus* collections, generalized estimating equations using a negative binomial model with log-link function was used to estimate the mean values and the difference in *Ae*. *albopictus* density between habitats for the different seasons, with date of collection, study area and time of collection included as factors (IBM SPSS statistics, version 20). The resistance status of *Ae*. *albopictus* was calculated using the WHO criteria. If > 10% mortality was observed in controls, the exposure data were corrected using Abbott’s formula [75]. Mortality of the exposed mosquitoes was calculated by summing the number of dead mosquitoes across all replicates and expressing this as a percentage of the total number of exposed mosquitoes (1) [72].

$$Observed\;mortality=\frac{Total\;number\;of\;dead\;mosquitoes}{Total\;exposed}\times 100$$ (1)

A population was considered resistant if the mortality after 24 hours was under 90%%, resistance was suspected when mortality was between 90 and 98% and a population was deemed susceptible when mortality was over 98%.

### Ethics

The use of the human-baited double net trap method was approved by the ethics committee of the Ministry of Health in Lao PDR (approval number 017/NECHR issued 21-04-2013) and the School of Biological and Biomedical Sciences Ethics Committee, Durham University (issued 25-07-2013). Human participants were not involved in any of the other activities. The field studies did not involve endangered or protected species.

## Results

### Seasonality and habitat preference of adult mosquitoes

**Fig 2:**
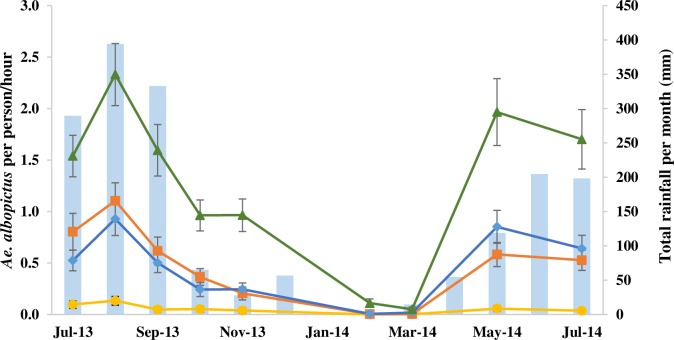
The average number of female *Aedes albopictus* collected per person per hour during the nine months of collection from July 2013 to July 2014 (▬▲▬ secondary forests, ▬♦▬ mature plantations, ▬■▬ immature plantations, ▬●▬ villages).

A total of 6,302 females and 887 males *Ae*. *albopictus* were collected during the study. Adult mosquitoes were collected throughout the 24 h collection period. Highest activity was during the daylight hours (6.00 to 18.00 h) with a mean of 0.64 (95% CI 0.61–0.68), compared to 0.17 (95% CI 0.15–0.18) at night. The numbers of *Ae*. *albopictus* were highly seasonal, with more than 90% (5,776/6,302) of the female *Ae*. *albopictus* collected during the rainy seasons, from July to October 2013 and from May to June 2014 (Fig 2). During the rainy seasons on average 0.88 (95% CI 0.83–93) *Ae*. *albopictus* were collected per hour during the daylight hours, which was 5.6 times higher (GEE *P* = 0.010, 95% CI 1.47–21.36) than in the dry season when 0.16 (95% CI 0.14–0.18) *Ae*. *albopictus* were collected.

There were few *Ae*. *albopictus* collected in villages during the rainy seasons, with an average of 0.04 females collected per person per hour (95% CI 0.03–0.06; Table 1). In contrast, collections were 48, 16 and 15 times higher in secondary forests, in immature plantations and in mature rubber plantations than in villages, respectively (Table 1). In the dry season few females were collected in the villages; an average 0.005 female *Ae*. *albopictus* (95% CI 0.00–0.01; Table 1). In contrast collections in the secondary forests collections were 93 times higher, in mature rubber plantations 26 times higher and in immature rubber plantations 17 times higher (Table 1).

**Table 1:** Multivariate analysis of habitat variability associated with female *Ae*. *albopictus* collected using human-baited double net traps during day-time from 06.00 to 18.00 h during the rainy season (April to October) and dry season (November to March).

Season	Habitat	n	Mean no. collected per person/hour (95% CI)		OR (95% CI)		*P*
Rainy	Secondary forest	2,701	2.08	(1.93–2.24)	48.53	(19.66–119.76)	<0.001*
	Mature rubber plantation	924	0.71	(0.64–0.78)	15.77	(6.61–37.65)	<0.001*
	Immature rubber plantation	898	0.69	(0.62–0.76)	16.00	(7.69–33.33)	<0.001*
	Village	57	0.04	(0.03–0.06)	1		
Dry	Secondary forest	281	0.43	(0.35–0.52)	93.30	(63.23–137.67)	<0.001*
	Mature rubber plantation	79	0.12	(0.09–0.16)	26.43	(21.95–31.82)	<0.001*
	Immature rubber plantation	51	0.08	(0.05–0.11)	17.08	(15.58–18.72)	<0.001*
	Village	3	0.005	(0.00–0.01)	1		

Results are shown using generalized estimating equations with odds ratio (OR) and 95% confidence interval (CI).

*significantly different, P<0.05

A similar habitat preference was seen for the male mosquitoes. In the villages the lowest number of male *Ae*. *albopictus* were collected, with an average of 0.013 samples collected per person per hour (95% CI 0.01–0.02) during daylight hours. In the secondary forests, male collections were 19 times higher (*P* < 0.001, 95% CI 12.38–27.72), with an average of 0.25 collection per person per hour (95% CI 0.21–0.28). In the mature rubber plantations collections were 9 times higher (*P* < 0.001, 95% CI 5.86–13.40), with an average of 0.12 *Ae*. *albopictus* mosquitoes per person per hour (95% CI 0.10–0.14). In the immature rubber plantations collections were 4 times higher (*P* < 0.001, 95% CI 2.80–6.66), with 0.06 males per person per hour (*P* < 0.001, 95% CI 0.04–0.07).

### Diel landing pattern

**Fig 3:**
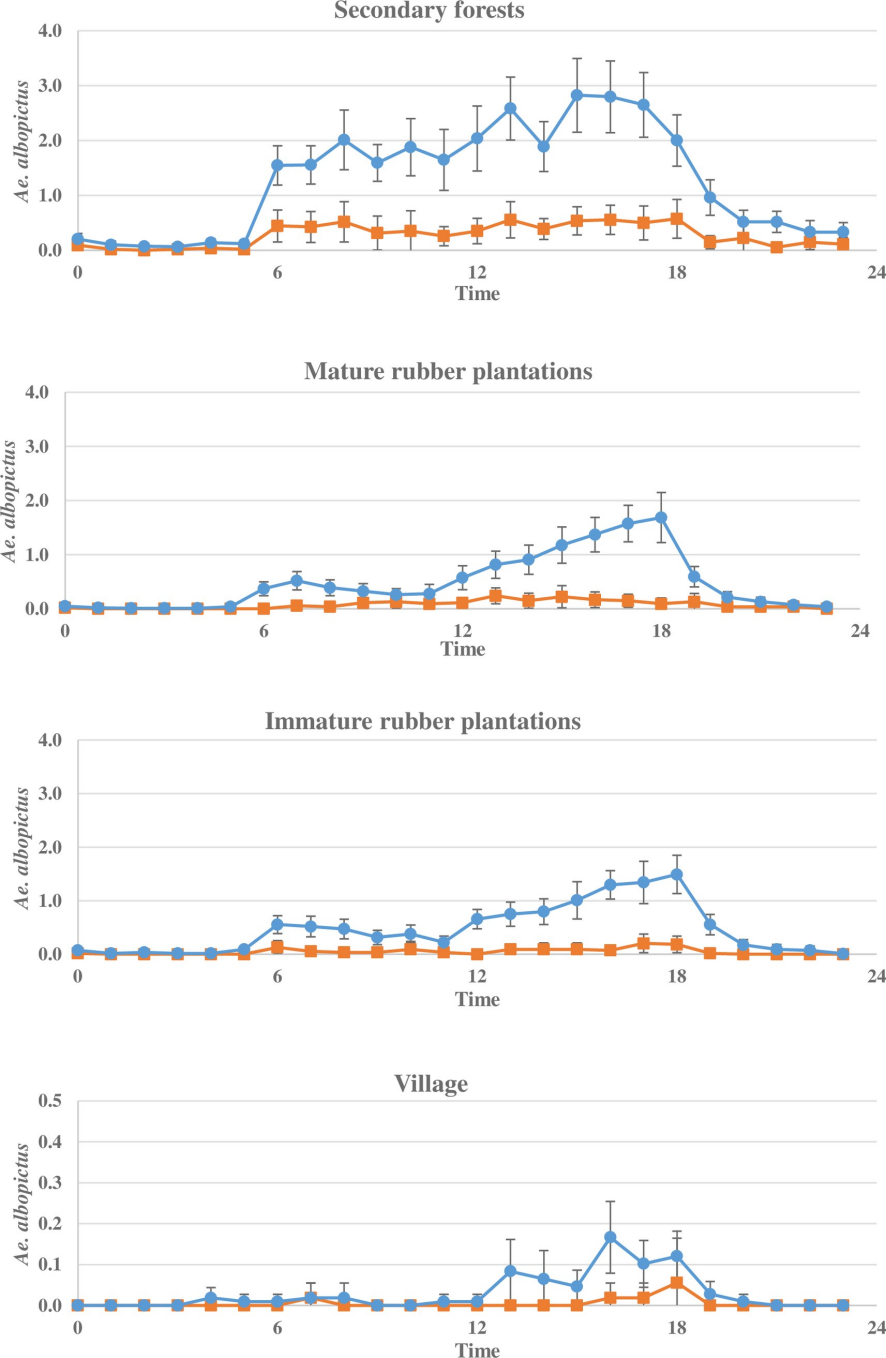
*Aedes albopictus* behaviour in the different habitats. The average number of female *Aedes albopictus* collected per person per hour in the secondary forests, mature plantations, immature plantations and villages during 24 h (**▬●▬** rainy season (April to October), **▬■▬** dry season (November to March)).

Host-seeking activity differed in the habitats between seasons. During the dry season, low and stable activity was found in all habitats (Fig 3). During the rainy season, the activity of mosquitoes generally peaked in the late afternoon. In the secondary forests activity was high during daylight hours with peak from 15.00 to 18.00 h, when between 2.0 and 2.8 females were collected per person per hour. In the mature and immature rubber plantations activity was low until 12.00 h, after which host-seeking activity increased. Peak activity was at 18.00 h when 1.69 and 1.49 *Ae*. *albopictus* were collected in mature and immature rubber plantations, respectively. In the villages there were few *Ae*. *albopictus* activity, with a small increase from 12.00 h to 18.00 h of 0.17 *Ae*. *albopictus*. Male *Ae*. *albopictus* displayed similar behaviour as the female mosquitoes with more than 95% of the male *Ae*. *albopictus* (846/887) collected during the day-time, building to a peak in the late afternoon from 16.00 to 18.00 h.

### Adult survival

A total of 1,048 females were dissected to determine parity. Overall parity was extremely high with 92% parous in the secondary forests (309/327), 91% parous in mature rubber plantations (406/447) and 87% parous in immature rubber plantations (234/269). Only five females were dissected for parity in the villages of which three were parous, making the estimation of parity uncertain.

### Molecular identification of arboviruses

A total of 7,189 *Ae*. *albopictus* mosquitoes (6,302 females, 887 males) were pooled in 1,252 tubes and tested. Whilst none displayed amplicon of the expected size for pan-alphaviruses RT-PCR, 36 pools displayed a positive signal when screened by the pan-flaviviruses; positives were found for both male (6.8%, 9/133) and female (2.4%, 27/1,119) pools. No RT-PCR signal for flavivirusus sequence could be found from pools from the village (0/30). However, 3.7% of *Ae*. *albopictus* pools from the mature rubber plantations (11/294), 2.9% of pools from the secondary forests (20/690) and 2.1% of pools from the immature rubber plantations (5/238) were found positive for pan-flavivirus sequences.

### Larval surveys

**Fig 4:**
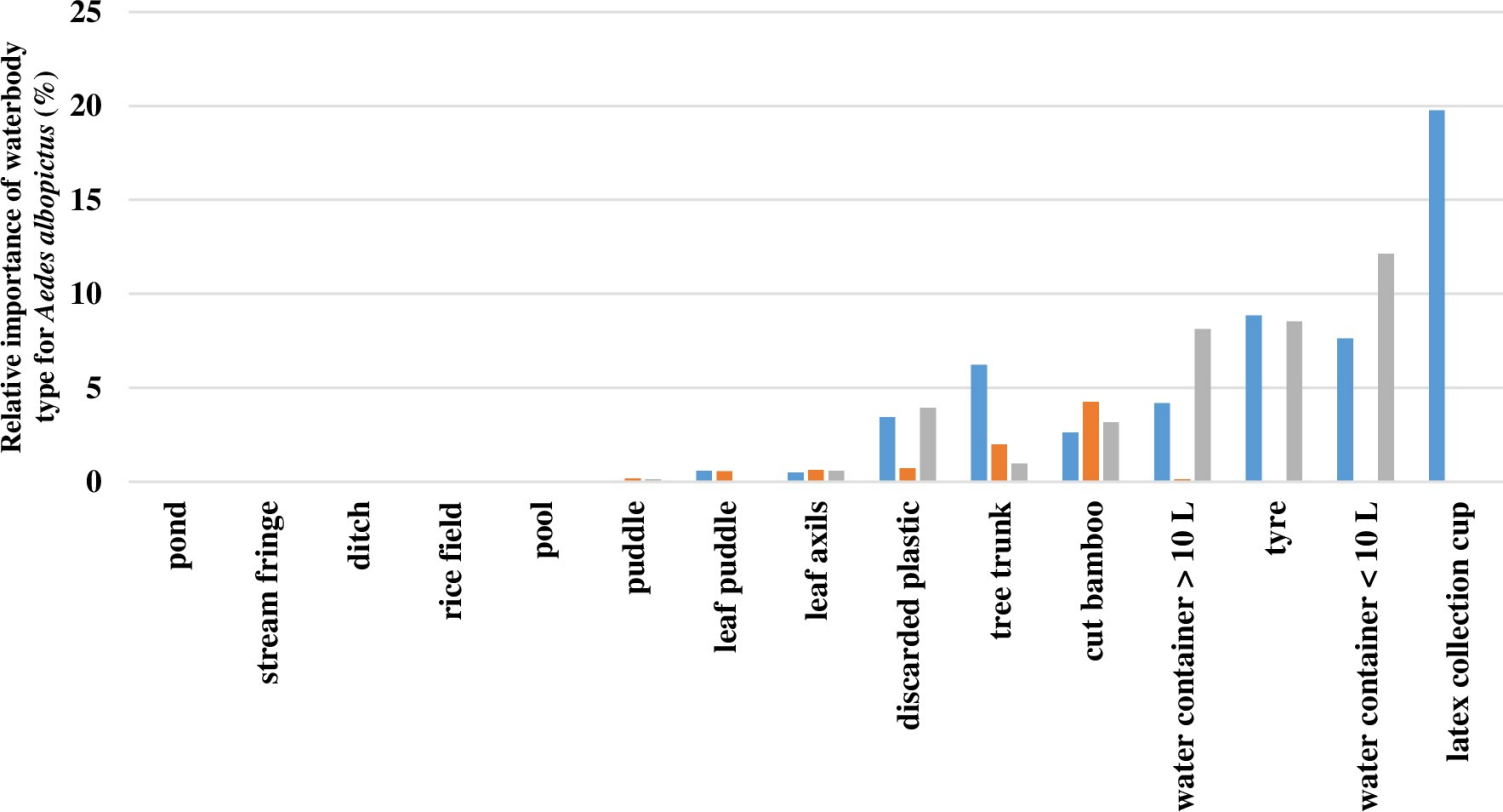
Relative importance of the waterbody types collected in villages (grey), mature rubber plantations (blue) and immature rubber plantations (orange) for the total number of emerged *Aedes albopictus*.

**Fig 5:**
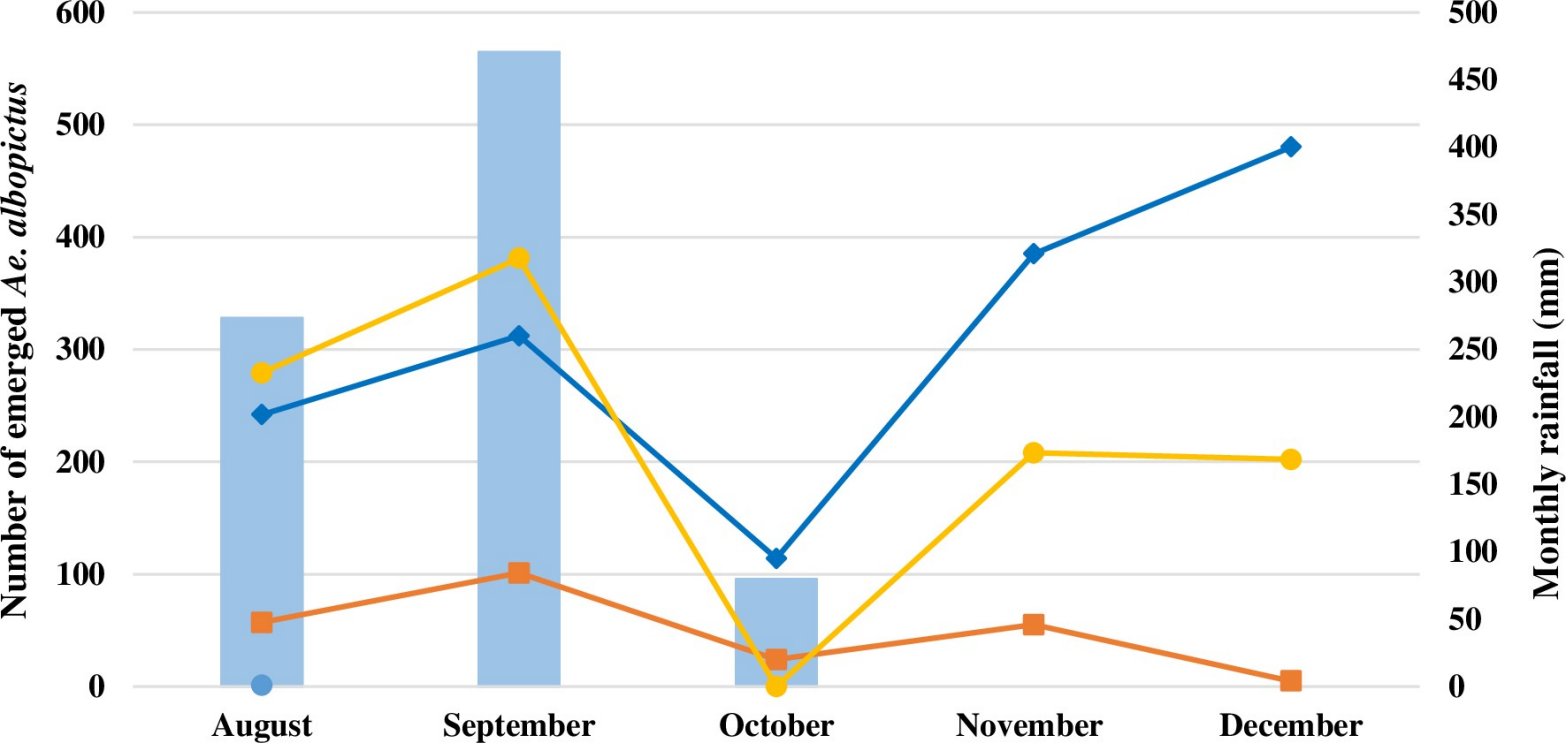
The total number of emerged *Aedes albopictus* per month in 2014 (▬●▬ villages, ▬♦▬ mature plantations, ▬■▬ immature plantations).

Between August and December 2014, 1,379 water bodies were surveyed of which 53% (724/1,379) contained mosquito larvae and/or pupae. Of the 11,468 immature *Aedes* collected, 3,757 adults emerged, of which 76% were *Ae*. *albopictus* (2,845/3,757). Most *Ae*. *albopictus* mosquitoes emerged from latex collection cups (20%, 562/2,845), small water containers (< 10L, 20%, 562/2,845) and tyres (17%, 495/2,845; Fig 4). Whilst few *Ae*. *albopictus* were collected in immature rubber plantations, higher numbers were collected in the villages and mature rubber plantations. This distribution was especially marked from August to September 2014 and from November to December 2014 (Fig 5).

Fifty-four percent of the *Ae*. *albopictus* were collected from the mature rubber plantations (1,533/2,845), with 37% of these collected in latex collection cups (562/1,533), 16% from tyres (252/1,533), 14% from small water containers (217/1,533) and 12% from tree trunks (177/1,533; Fig 4). Thirty-eight percent of the *Ae*. *albopictus* were found in villages (1,070/2,845), of which 32% were found in water containers < 10 L (345/1,070), 23% in tyres (243/1,070) and 22% in water containers > 10 L (231/1,070; Fig 4). In the immature rubber plantations, 242 *Ae*. *albopictus* emerged, of which 50% were collected from cut bamboo (121/242) and 24% from tree trunks (57/242; Fig 4).

### Insecticide resistance

#### Adult bioassays

For all bioassays, mortality in the control tubes never exceeded 10% so no correction was necessary. In Luang Prabang province, the three *Ae*. *albopictus* populations tested were all resistant to DDT and malathion, with mortality 24 hours after exposure ranging from 27 to 78% (Table 2). Similarly, in Vientiane city the four populations tested were all resistant to DDT, with mortalities ranging between 27 and 90%. Three strains from Vientiane were also resistant to malathion with mortality ranging between 20 and 57%. Only one strain from Suanmone village in Vientiane was susceptible to malathion. All *Ae*. *albopictus* samples tested from Vientiane and Luang Prabang were susceptible to deltamethrin and permethrin with 100% mortality, apart from one population in Kao-gnot, Vientiane-city where resistance to permethrin was suspected with 96% mortality.

**Table 2:** Resistance status of adult *Aedes albopictus* to DDT, malathion, deltamethrin and permethrin according to WHO criteria [72, 74, 76].

Province	District	Village	Insecticide	n	% Mortality	Status
Vientiane capital	Sisattanak	Kao-gnot	DDT	95	63	Resistant
			malathion	100	20	Resistant
			deltamethrin	95	100	Susceptible
			permethrin	102	96	Suspected
	Sisattanak	Suanmone	DDT	78	90	Resistant
			malathion	75	100	Susceptible
			deltamethrin	96	100	Susceptible
			permethrin	75	100	Susceptible
	Xaysettha	Sengsavang	DDT	95	27	Resistant
			malathion	98	52	Resistant
			deltamethrin	87	100	Susceptible
			permethrin	100	100	Susceptible
	Xaythany	Oudomphon	DDT	79	58	Resistant
			malathion	70	57	Resistant
			deltamethrin	72	100	Susceptible
			permethrin	68	100	Susceptible
Luang Prabang	Luang Prabang	Nasangveuy	DDT	102	27	Resistant
			malathion	99	86	Resistant
			deltamethrin	94	100	Susceptible
			permethrin	99	100	Susceptible
	Luang Prabang	Phonphao	DDT	85	48	Resistant
			malathion	68	51	Resistant
			deltamethrin	82	100	Susceptible
			permethrin	73	100	Susceptible
	Nan	Huayhoy	DDT	76	78	Resistant
			malathion	73	49	Resistant
			deltamethrin	53	100	Susceptible
			permethrin	82	100	Susceptible

Diagnostic Doses (DD) used; 4% DDT, 0.8% malathion, 0.05% deltamethrin and 0.25% permethrin

#### Larval bioassays

For all bioassays, mortality in the control tubes never exceeded 10% so no correction was necessary. All larval *Ae*. *albopictus* populations tested were highly resistant to DDT with mortality ranging from 3 to 44%, except in Oudomphon where resistance was suspected (98% mortality; Table 3). In Luang Prabang province, moderate resistance to temephos was suspected in Phonphao village (92% mortality) and the population from Huayhoy village showed resistance with 74% mortality. In Vientiane capital, samples from both Suanmone and Oudomphon were resistant to temephos with 42% and 87% mortality, respectively. The samples from Sengsavang were susceptible to temephos and in Kao-gnot population resistance was suspected (92% mortality). In both Luang Prabang and Vientiane provinces, (suspected) resistance to deltamethrin was observed with mortality ranging from 6 to 99%. All the populations tested against malathion and permethrin were susceptible, except for Sengsavang where resistance to permethrin was suspected.

**Table 3:** Resistance status of *Aedes albopictus* larvae against DDT, temephos, malathion, deltamethrin and permethrin.

Province	District	Village	Insecticide	n	% Mortality	Status
Vientiane capital	Sisattanak	Kao-gnot	DDT	100	3	Resistant
			temephos	100	92	Suspected
			deltamethrin	100	93	Suspected
			permethrin	100	100	Susceptible
	Sisattanak	Suanmone	DDT	150	28	Resistant
			temephos	125	42	Resistant
			malathion	200	100	Susceptible
			deltamethrin	100	82	Resistant
			permethrin	150	100	Susceptible
	Xaysettha	Sengsavang	DDT	100	44	Resistant
			temephos	100	99	Susceptible
			deltamethrin	100	99	Susceptible
			permethrin	100	95	Suspected
	Xaythany	Oudomphon	DDT	50	98	Suspected
			temephos	200	87	Resistant
			malathion	200	100	Susceptible
			deltamethrin	50	94	Suspected
			permethrin	50	100	Susceptible
Luang Prabang	Luang Prabang	Nasangveuy	DDT	100	85	Resistant
			temephos	100	98	Suspected
			deltamethrin	100	6	Resistant
			permethrin	100	100	Susceptible
	Luang Prabang	Phonphao	DDT	150	11	Resistant
			temephos	200	92	Suspected
			malathion	200	100	Susceptible
			deltamethrin	150	91	Suspected
			permethrin	200	100	Susceptible
	Nan	Huayhoy	DDT	145	17	Resistant
			temephos	100	74	Resistant
			malathion	200	100	Susceptible
			deltamethrin	150	90	Suspected
			permethrin	150	100	Susceptible

Diagnostic doses uses were 0.04 mg/L (DDT), 0.0132 mg/L (temephos), 1mg/L (malathion), 0.00132 mg/L (deltamethrin) and 0.0014 mg/L (permethrin)

## Discussion

Although individual studies on *Ae*. *albopictus* adult host seeking behaviour, larval habitats and insecticide resistance status of *Ae*. *albopictus* have been conducted [3, 5, 7, 29, 77–80], this is the first paper that combines all three topics. In concurrence with other studies throughout the world, most mosquito host seeking activity occurred in daylight hours, with an increase after dawn and peaking in the late afternoon [28, 77, 78, 81]. In this study, we demonstrate the forested nature of disease transmission, with high numbers of adult females being attracted to people in the natural and man-made forests with only a few adult mosquitoes found in the villages. The insecticide bioassays revealed a similar pattern of resistance in rural and urban areas. Resistance to malathion and DDT was identified in adult populations, and temephos resistance in larvae. Pyrethroid resistance was not detected in rural nor in urban populations.

*Aedes albopictus* prefers densely vegetated habitats, where human densities are generally low and irregular compared to urban areas [3, 5, 7, 29]. The risk of *Ae*. *albopictus* exposure was between 15 and 93 times higher in the forested areas than in the village. The highest numbers of adult mosquitoes were collected in secondary forests, their primordial habitat. This habitat is typical for *Ae*. *albopictus* throughout SEA, presumably because of the highly vegetated under storey, high relative humidity, high shade and moderate temperatures [7, 36, 82–85]. High numbers were also found in immature and mature rubber plantations. Colonisation of rubber plantations by *Ae*. *albopictus* has also been recorded in other parts of SEA before [86–88], presumably because it provides a habitat similar to natural forests; being shaded, with moderate temperatures and many aquatic habitats. The low numbers of adult mosquitoes collected in the villages and high abundance in forests habitats is typical of this species [7, 28, 29, 82, 84, 89]. This drastic difference of vector densities and mosquito behaviour in rural areas of Lao PDR strongly suggest that the risk of exposure to vector borne diseases could be significantly higher during forestry activities. This emphasizes the need to expand control efforts from the villages to the forest habitats. This is especially important for the rubber plantations, where in the rainy season regular human activity takes place [90]. It was surprising to find high numbers of immature stages in the villages in the year following the adult collections. This suggests that there are large variations in abundance in villages between years.

Screening of mosquitoes’ specimen by RT-PCR revealed the presence of flavivirus sequences both in males and females collected from the forest and rubber plantation habitats. Since the degenerated primers used may also match to insect specific flavivirus, the sequences detected here do not necessarily correspond to virus infective and pathogenic for humans. Our results suggest that people present in the forest habitats have a higher flaviviruses exposure risk than people in the villages, due to the high density of *Ae*. *albopictus* and the presence of flaviviruses. The absence of flaviviruses in the villages is possibly related to the low number of *Ae*. *albopictus* collected in this habitat. Further studies are necessary to understand the dynamics of flaviviruses in the villages.

Male *Ae*. *albopictus* activity was almost identical to females, with activity increasing during daylight and peaking in the late afternoon. Similar behavioural patterns have been identified for *Ae*. *albopictus* in other parts of the world [77, 78, 91, 92]. Almost 7% of the adult *Ae*. *albopictus* males collected using human-baited double net traps, displayed flavivirus sequences. Since males do not blood-feed, the presence of these flavivirus sequences could be the result of vertical transmission, from parent to offspring. As vertical infection of dengue viruses are suggested to be low in the field [93], the high rate of vertical transmission identified suggests the presence of insect flaviviruses infestations [94, 95]. Testing adult males collected in the field may increase the sensitivity of molecular vector surveillance and could be used as an early alert method.

Although *Ae*. *albopictus* originates from forests, where the immature stages are found in aquatic habitats such as tree holes, bamboo stumps, and bromeliads [28, 96], it has readily adapted to breeding in man-made containers. In our larval survey most of the immature stages were from the mature rubber plantations, where they were found in latex collection cups, tyres and small water containers. Other studies in India, Malaysia and Thailand have also shown that mature rubber plantations are highly productive sites for *Ae*. *albopictus* [86–88, 97, 98]. In the villages water containers and tyres both were important aquatic habitats, as has been reported previously from central Lao PDR [31] and other parts of SEA [28, 29, 64, 99–103].

High levels of resistance to DDT was found in both the larval and adult populations surveyed. Even though DDT is not used for vector control today, DDT resistance can last for decades and result in cross-resistance to pyrethroids [104–106]. Although resistance to both DDT and pyrethroids were not identified within a single population during the adult bioassays, the larval bioassays did identify several populations within which mosquitoes were less sensitive to both DDT and a pyrethroid. Further investigation is needed to understand the mechanism behind the resistance, including possible cross-resistance. Since permethrin is incorporated into many long-lasting insecticidal nets, currently a central pillar for vector control, the banning of DDT in both agriculture and vector control should be strictly upheld. The organophosphate malathion, used for vector control in the 1990s, should be used with caution, because of the high levels of resistance detected in six of the seven adult mosquito populations tested. With dengue, chikungunya and possibly Zika expected to result in more morbidity in the region in the next decade, it is of importance to establish routine monitoring of insecticide resistance in this species.

The important limitation of insecticide resistance studies are that the resistance statuses measured using bioassays have not been directly related to the failure to control *Ae*. *albopictus* in the field. Thus additional surveys are necessary to understand the impact of resistance development on vector control. Furthermore, the diagnostic doses (DD) used to test the susceptibility of adult *Ae*. *albopictus* in this study are the same as those used for *Ae*. *aegypti*. This may not have been accurate, as a study in Thailand showed that *Ae*. *albopictus* might need a lower DD for deltamethrin than the DD recommended by the WHO for *Ae*. *aegypti* (i.e. 0.026% compared to 0.05%) [107]. Furthermore, recently WHO changed the DD for permethrin from 0.05% to 0.03% [76]. As the DD used in this study were sometimes higher than the DD suggested by WHO and literature, it is possible that we underestimated the levels of resistance in our populations.

The control of outdoor-biting mosquitoes is challenging since the most effective vector control tools are directed at those species that enter houses. Personal protection methods should be recommended for those visiting the secondary forests and when working in the rubber plantations. People could wear long-thick trousers, long-sleeved shirts and closed shoes with high socks to minimize skin exposure to mosquitoes. To decrease nuisance of mosquitoes, repellents applied to exposed skin or clothing could be recommended. Research, however, is needed to identify the best outdoor personal protection method or a combination of methods, to reduce biting and decrease disease incidence. These methods should be adapted to the local working conditions, to ensure there is high social acceptability and high user compliance. For those resting in forests or rubber plantations, the use of a long-lasting insecticidal net would offer protection.

To control both females and males, which are important for vertical transmission, larval control should be implemented in the villages and rubber plantations. Larval control in the forest areas will likely not be effective due to the high density of breeding sites and the difficulty in reaching a large number of sites. In rubber plantations, latex collection cups need to be turned upside down when not in use for more than one week and stored in shelters during the dry season when latex is not tapped. In both habitats water containers need to be covered and tyres removed. Throughout the year, households should be encouraged to weekly clean in and around their houses and empty all waterbodies. If water containers cannot be removed, covered or regularly emptied, the larvicide Abate® or a biological control can be used. As moderate resistance to the insecticide temephos, the active ingredient of Abate®, was found, its implementation should be properly managed and resistance status closely monitored. Biological control entails the introduction of organisms that reduce the population of the target species. Larvivorous fish species such as *Gambusia* spp. and *Poecilia reticulate* or predacious arthropods such as *Toxorhynchites splendens* or the copepod *Mesocyclops* could be released in large water bodies [108–110]. The bacteria *Bacillus thuringiensis var*. *israeliensis* (*Bti*) can also be used to treat both large and small water containers [111].

## Conclusion

Vector control is currently the most effective way to fight against vaccine-orphan viral vector-borne diseases. Yet, the identification of proper control methods has been challenging due to the variable bionomics of *Ae*. *albopictus* and limited knowledge on its resistance status. This study has highlighted the variability of *Ae*. *albopictus* presence in rural areas and the importance to include rubber plantations and secondary forests in control efforts. Personal protection is especially important in the secondary forests and rubber plantations during the day, when a high density of the vector species was identified. Elimination of the water bodies in latex collection cups is important for reducing the aquatic stages of development in the rubber plantations. Additionally, tyres and water containers are important to control in both the villages and rubber plantations. Adult control can incorporate pyrethroids in its methods, while the popular larvicide temephos should be used more cautiously. This study demonstrates that rural areas in northern Laos are potential hot spots for arboviral disease transmission and that vector control should be enhanced in this area.

## Supporting information

S1 Table Description of the waterbody habitats. (DOCX)
