# Supplementary material for: Bionomics and insecticide resistance of the arboviral vector Aedes albopictus in northern Lao PDR
Source: PLoS One. 2018 Oct 25;13(10):e0206387. doi: 10.1371/journal.pone.0206387 (PMC6201963; doi:10.1371/journal.pone.0206387)
Supplement: S1 Table — (DOCX) [file pone.0206387.s001.docx]

**Supplementary table 1; Description of the waterbody habitats**

| **Waterbody habitat** | **Description** |
| --- | --- |
| Cut bamboo | Both still growing and used for construction of gates, pig stalls and chicken houses |
| Leaf axils | Axils of a banana tree |
| Discarded plastic | Including broken shoes, plastic bottles and plastic bags |
| Water container < 10 L | Generally containers used to transport water, such as buckets |
| Water container > 10 L | Generally containers used to store water, including drums and cement tubs |
| Puddle | Small (< 2 m diameter) and shallow (< 50 cm) standing water body with water originating from rainwater, usually drying out toward the end of the rainy season |
| Pool | Generally medium sized (2-5 m diameter) standing water body with water originating from ground water and rainwater |
| Pond | Permanent large (> 5 m) water body with water originating from both ground water and rain water |
| Stream fringe | Fringe of year-round stream |
| Latex collection cup | Both containing and not containing latex |
| tree trunk | Holes in the trunk of a tree |
| Ditch | A narrow channels (< 2 m diameter) dug at the side of a road or around a house |
| Leaf puddle | Fallen leaves from different plants that provide small (< 20 cm diameter) temporary pockets of water |
| Tyre | An unused tyre |
| Rice field | Seasonally flooded areas used to grow lowland rice |
